# States’ racial resentment correlates with administrative distancing and lower rates of health plan selection in affordable care act marketplaces: a cross sectional analysis

**DOI:** 10.1186/s12913-023-10252-w

**Published:** 2023-11-01

**Authors:** Lonnie R Snowden, Genevieve Graaf

**Affiliations:** 1 Health Policy and Management, School of Public Health, University of California, Berkeley, Berkeley, CA, USA; 2 School of Social Work, University of Texas, Arlington 211 S. Cooper St, Arlington, TX 76019 USA

**Keywords:** Affordable Care Act, Healthcare marketplaces, State health policy, Racial resentment

## Abstract

**Background:**

In the United States, the Affordable Care Act (ACA) pursued equity in healthcare access and treatment, but ACA implementation varied, especially limiting African Americans’ gains. Marketplaces for subsidized purchase of coverage were sometimes implemented with limited outreach and enrollment assistance efforts. Reflecting state’s ACA receptivity or reluctance, state’s implementation may rest on sociopolitical stances and racial sentiments. Some states were unwilling to provide publicly supported healthcare to nonelderly, non-disabled adults— “the undeserving poor” —who evoke anti-black stereotypes. The present study assessed whether some states shunned Affordable Care Act (ACA) marketplaces and implemented them less vigorously than other states, leading to fewer eligible persons selecting insurance plans. It assessed if states’ actions were motivated by racial resentment, because states connote marketplaces to be government assistance for unworthy African Americans.

**Methods:**

Using marketplace and plan selection data from 2015, we rated states’ marketplace structures along a four-level continuum indicating greater acceptance of marketplaces, ranging from states assuming sole responsibility to minimal responsibility. Using national data from a four-question modern racism scale, state-wide racial resentment estimates were estimated at the state level. Analysis assessed associations between state levels of racial resentment with states’ marketplace structure. Further analysis assessed relationships between both state levels of racial resentment and states’ marketplace structure with states’ consumer plan selection rates—representing the proportion of persons eligible to enroll in insurance plans who selected a plan.

**Results:**

Racial resentment was greater in states with less responsibility for the administration of the marketplaces than actively participating states. States higher in racial resentment also showed lower rates of plan selection, pointing to less commitment to implementing marketplace provisions and fulfilling the ACA’s coverage-improvement mission. Differences persisted after controlling for differences in conservatism, uninsurance, poor health, and rejection of Medicaid expansion.

**Conclusions:**

Resentment of African Americans’ purported irresponsibility and entitlement to government assistance may interfere with states structuring and operating marketplaces to maximize health insurance opportunities for everyone available under the ACA.

**Trial registration:**

N/A.

Passage and implementation of the Affordable Care Act (ACA) were vigorously contested. Twenty-six states joined an unsuccessful lawsuit declaring the ACA unconstitutional and nineteen states proposed or adopted anti-ACA resolutions (NCSL 2013) [1]; the Republican-controlled Houses of Representative voted more than 50 times to repeal it [2]. Ten states currently reject Medicaid expansion but, during ACA implementation in 2014, 19 states rejected expansion [3].

ACA-initiated health insurance marketplaces, for purchase of publicly-subsidized private coverage (French et al., 2016), also stimulated great resistance. Marketplaces were rejected both by politically opposed individuals who refused to participate [4, 5] and by ACA-disapproving states. Many of these states declined the option for state-operated marketplaces, relying instead on the default federal marketplace to meet ACA mandated responsibilities. Public subsidies too were resisted: In a court challenge to ACA subsidies, many states with federally facilitated marketplaces sought denial of subsidies [6].

Many sociopolitical and historical factors drove objections to marketplaces and other ACA components [7], but racism played a prominent role [8]. African American President Obama’s sponsorship of the ACA earned it the nickname “Obamacare” and encouraged race-based adverse responses [9]. For example, states with proportionally higher African American populations rejected Medicaid expansion [3, 8, 10] and states with greater racial bias made available fewer ACA-stimulated Federally Qualified Health Centers [11]. Individuals higher in racial antipathy considered the ACA to have worsened healthcare, despite the ACA’s improving healthcare access and population health [12].

Marketplace and subsidy resistance are perceived as severing health insurance coverage from employment, violating a norm whereby health insurance rewards workers for holding socially valued occupations [13]. Unmistakably linking insurance coverage and personal worthiness and likely speaking for many supporters, former President Trump proclaimed: “Where I come from, you have to prove your worth. You have some guy with no college degree working a minimum wage job; no ambition, no goals, nothing to show for it. Yet for some reason, the current administration believes he — and millions of people like him, should have access to health insurance. It’s outrageous” [14]. This “just world” thinking [15], conceiving of health insurance coverage as a privilege not a right [16], also motivates Medicaid work requirements [17, 18].

“Personal worthiness” connotations are reinforced by marketplaces’ government sponsorship and by the fact that subsidies provide means tested government support. Both suggest “welfare” and can arouse anti-African American sentiments [3]. Anti-African American stereotypes include welfare dependency: welfare recipients are seen as African Americans in laboratory studies [19]. Marketplaces rely on private sector competition and consumer choice but racial resentment can target “unworthy” Africa Americans considered primary beneficiaries [20]. Labor market and income realities do dictate disproportionate African American benefit: lower occupational standing denies African Americans employment providing health insurance coverage as a fringe benefit [21] and African Americans’ lower incomes qualifies them more for financial support. Thus, 86% of uninsured African Americans applying through marketplaces qualified for subsidies [22].

Perceptions of African Americans’ disproportionate benefit from public programs can stimulate “racial resentment” [23]—a racially charged and politically consequential grievance associated with disapproval of policies seen as unfairly benefiting African Americans. Racial resentment is “symbolic racism’ [24], and recent conceptions [25] propose that it violates “just world” convictions [15], whereby economic and vocational success bespeaks worthiness. Such thinking can justify viewing African Americans disadvantaged social and economic standing as due to personal and cultural failings [25], not to well-documented, legitimate historical and contemporary roadblocks [26–31]. As greater racial resentment supports repealing the ACA [25], so might it also reinforce reluctance to sponsor marketplaces and, beyond marketplace structural choices, interfere with vigorously mounting outreach and enrollment efforts.

We investigated racial resentment’s association with states shunning or embracing marketplaces and marketplaces’ success in providing coverage to uninsured persons during early years of full implementation. We tested two hypotheses. The first was that states with greater racial resentment would take less responsibility for marketplaces along a continuum of responsibility. The second hypothesis was that states with greater racial resentment would have lower marketplace enrollment rates. We controlled for potentially confounding covariates.

## Methods

### Independent variable: racial resentment

The four-question modern racism scale (Kinder and Sanders, 1996) is used in political surveys and analyzed in election science survey data [27], and is perhaps the most widely used measure of symbolic racism (Enders, 2021). It consists of the following four questions answered on a 5-point Likert scale:

1. Over the past few years, blacks have gotten less than they deserve.
2. Irish, Italian, Jewish, and many other minorities overcame prejudice and worked their way up. Blacks should do the same without any special favors.
3. It’s really a matter of some people not trying hard enough; if blacks would only try harder, they could be just as well off as whites.
4. Generations of slavery and discrimination have created conditions that make it difficult for blacks to work their way out of the lower class.

Questions #1 and #4 are reverse scored such that higher scores indicate more racial resentment.

To estimate state populations’ racial resentment from surveys designed to represent the U. S. population, not states’ populations, investigators [31] used multilevel regression with post-stratification weighting [32]. They predicted, in multilevel models, individuals’ survey-expressed preferences or opinions at a first level of analysis from pertinent individuals’ characteristics (e.g., race, age). Using these estimates, they predicted at a higher-level the same preferences or opinions from pertinent state characteristics. Predicted values were then computed for every category of personal characteristic, cross-classified with every other category, creating “types” (e.g., African American males and females, white males and females). The values were then combined after weighting to reflect each state’s census-determined representation of persons in each category.

We downloaded state estimates provided by racial resentment investigators [31] from whom state estimates are available at 4-year intervals: including 2012, closest to marketplace inauguration in 2013. Accordingly, 2012-assessed racial resentment served as the key independent variable in models for predicting marketplace structure. As 2016-assessed racial resentment was closest to the 2014–2015 s open enrollment period—from which outcome data was drawn—racial resentment assessed in 2016 served as the key independent variable in models predicting plan selection rates.

### Dependent variables: marketplace structure and insurance plan selection rates

#### Marketplace structure

Reactions to ACA marketplace requirements presented a polarized response. Some states actively resisted marketplaces [33]: they declined and even returned federal grant money for marketplace planning, sometimes by passing laws and constitutional amendments banning state-run exchanges. Other states resisted passively, disregarding opportunities to establish state-based exchanges [33].

Other states maximized responsibility for state control to reduce uninsurance rates, largely at federal expense, to enhance marketplace performance. They accepted planning grants and secured supplemental funding for advertising, outreach, and enrollment assistance and they maximized state control of required marketplace functions in order to strengthen the marketplace’s recruitment efforts [33]. A few states seeking greater state control relied on the healthcare.gov platform for eligibility screening and enrollment but most relied state-run platforms for screening and enrollment. Often, sometimes hard-to-reach ethic minority communities were a particular focus of effort [34].

In 2015, the ACA allowed states to implement one of three marketplace structures [35]: State Run Exchanges, Federal State Partnerships and Federally Facilitated Exchanges [36]. In State Run Exchanges, some states relied on the federal platform for eligibility determination and enrollment but assumed all other marketplace responsibilities themselves. Because these states held less responsibility than fully State-Run marketplaces, we assigned them a separate rating: State-based Marketplace-Federal Platforms. The four resulting categories were coded to reflect increasing state responsibility:

0. Federal Marketplaces (Federally Facilitated Exchanges): The federal government assumes sole responsibility for marketplace operations. Through HealthCare.gov, people shop for and enroll in through websites, call centers, and in-person help. The federal marketplace determines eligibility, allows consumers to compare prices, learn if they qualify for subsidies, and processes enrollment. (n = 27)
1. Federal-State Marketplace (State-Federal Exchanges): States administer selected management functions, often those preserving powers of state insurance commissioners such as certification of plans, monitoring and regulatory control; or they select marketplace functions (e.g., outreach, enrollment assistance) for state responsibility and oversight. The federal government conducts eligibility determination and enrollment through HealthCare.gov. (n = 7)
2. State-based Marketplace-Federal Platform. States conduct all marketplace activities, (e.g., outreach, enrollment assistance, oversight of participating health plans). The federal government conducts eligibility determination and enrollment through the federal website, HealthCare.gov. (n = 5)
3. State-based Marketplace (State-Run Exchanges): States conduct all marketplace activities and conducts eligibility determination and enrollment through state-operated websites. (n = 11)

We downloaded 2015 state designations from the National Conference of State Legislators Report [36]. To match our dating of plan selection rates (see below), we identified State-based marketplaces for 2015 using the federal platform [33] and entered them accordingly.

#### Plan selection rates

Plan selection is newly selecting a plan, reenrolling in a plan, or switching plans [37]. Plan selection rates are calculated for states as a proportion of persons eligible to enroll at the end of the open enrollment period. We used selection rates at the second open enrollment period, November 15, 2014 to February 15, 2015, drawn from Kaiser Family Foundation Reports. We chose second rather than the first open enrollment to allow greater marketplace stabilization and resolution of start-up problems. Kaiser Family Foundation downloads the data from Office of the Assistant Secretary for Planning and Evaluation (ASPE), Department of Health and Human Services’ Health Insurance Marketplace Open Enrollment Reports. They report each state’s rate at the end of the second open enrollment period: November 15, 2014 to February 15, 2015.

### Control variables

Racial resentment is more prevalent in states with larger African American populations [38] and, as documented below, states with greater racial resentment show more of factors correlated with marketplace preferences and performance: sociopolitical conservatism, poor health and higher uninsurance rates, and more marketplace eligible people because some states rejected Medicaid expansion. We assessed these characteristics in 2013, preceding or coinciding with early-stage marketplace operations.

#### Conservatism

Critics sometimes contend that the racial resentment scale confounds race-based disapproval with non-racial conservatism’s commitment to personal responsibility and insistence on a small government providing limited economic assistance [39]. Although complexities remain [29], conservatism appears to play only a limited role in explaining responses to the racial resentment scale [28, 40].

However, our assessment uncovered notable state-level association between racial resentment and conservatism. Because states’ racial resentment proved strongly correlated with greater conservatism (rho = .50, p < .01) we controlled for conservatism. In Gallup’s 2013 tracking poll on a large, nationally representative sample, respondents were asked to describe their political views as ‘liberal,” “moderate,” or “conservative” [41]. We entered each states’ percent of people rating their views as conservative.

#### Uninsurance

Uninsured individuals have incentives to seek coverage to avoid out-of-pocket health care expenditures and maintain better health [42, 43]. States with higher African American populations—and greater racial resentment– have higher uninsurance rates [44]. In our assessment racial resentment proved significantly associated with uninsurance rates (rho = 0.32, p > .05).

To control for uninsurance, we focused on potential purchasers of marketplace policies, nonelderly adults. We excluded children because they are covered under pre-ACA Medicaid and by the Children’s Health Insurance Program (CHIP) and older adults who are covered by Medicare. From the Census Bureau’s American Community Survey, The Kaiser Family Foundation calculates uninsurance rates by state for nonelderly adults, ages 19–64 [45]. We downloaded 2013 uninsurance rates for each state.

#### Poor health

Poor self-rated health correlates strongly with greater healthcare utilization and mortality [46] and unhealthy individuals seek insurance coverage to pay for treatment of their untreated illnesses (“adverse selection”) [47]. At the state level, to avoid bearing expenses from uncompensated treatment [48], states with less healthy populations have an incentive to maximize ACA coverage opportunities.

In our assessment, racial resentment indeed correlated with proportions of states’ residents reporting poor health (rho = 0.46, p > .01) and we controlled for it accordingly. To measure state populations’ health, we used the Behavioral Risk Factor Surveillance System (BRFSS), a state-based, random-digit-dialed telephone survey with about 400,000 adult respondents aged 18 years and older per year. Respondents are asked whether “in general” their health is excellent, very good, good, fair, or poor. Kaiser Family Foundation reports each state’s proportion of nonelderly persons reporting poor health [49]. We downloaded each state’s 2013 proportion of nonelderly poor person who rate themselves in poor health.

#### Rejection of medicaid expansion

Medicaid coverage was denied to residents in expansion-rejecting states unless they qualified under old and restrictive rules for Medicaid eligibility [50]. Yet through marketplaces, states could offer subsidized private coverage to many persons denied expanded Medicaid and more people qualified for subsidized marketplace coverage in expansion-rejecting than in expansion-accepting states [51]. Accepting Medicaid expansion proved negatively correlated with racial resentment as a trend (rho = -0.25, p > .08) and we controlled for states’ acceptance of Medicaid expansion as of 2014. The Urban Institute lists states that did and did not expand Medicaid when expansion began in 2014 [52]. We coded states as follows: accepting = 1, rejecting = 0.

### Analysis

To test our first hypotheses, we regressed on each state’s marketplace structure racial resentment scores and covariates. To test our second hypothesis, we regressed racial resentment and covariates on states’ rates of persons selecting a market insurance plan. Because our second hypothesis concerned racial resentment’s impact beyond effects associated with marketplace structure (as were assessed in the first model), in estimating the association between racial resentment and plan selection rate, we controlled for marketplace structure along with other covariates. In both models, we employed ordinary least squares (OLS) estimation with robust standard errors to guard against departures from assumptions, including homoskedasticity.

To check the sensitivity of results, we repeated both analyses in two ways. We retested our first hypothesis using racial resentment measured beyond initial marketplace implementation, in 2016 instead of 2012. We retested our second hypothesis in 2012 instead of 2016, using racial resentment measured earlier than the second enrollment period. In a second round of checks, to assess whether OLS misrepresents marketplace findings, we retested the marketplace model with Generalized Least Squares (GLS) with a Probit link. GLS relaxes OLS assumptions and Probit relaxes linear scaling expectations for marketplace structure. We retested plan selection rates after logarithmic transformation to negate the impact of possible outliers that might have distorted results.

## Results

Table 1 presents descriptive statistics on state policy implementation and political characteristics. Twenty-seven states operated federal marketplaces only, seven were state-federal partnerships, five were state-based with federal platforms, and 11 were state-based (M = 1.0, SD = 1.25) marketplaces. States’ average plan selection rate was 38.60%. Racial resentment scores in 2016 averaged 0.633 (SD = 0.032) (SD = 11.29%). States’ average percentage of politically conservative residents was 38.45% (SD = 6.15%) and uninsurance rates for adults, ages 19–64, averaged 19.07% (SD = 5.36%). In 2014, nineteen states had rejected Medicaid expansion and thirty-one had accepted.

**Table 1:** Descriptive Statistics: State’s Marketplace Structure, Racial Resentment, Conservatism, Uninsurance, Poor Health, Medicaid Expansion

Dependent Variables	Mean	SD
Marketplace Structure (0 = federal, 3 = state) Plan Selection Rate	1.00 38.60%	1.25 11.29%
**Independent Variables**		
Racial Resentment	0.633	0.032
Conservative	38.45%	6.15%
Adult Uninsurance Rate	19.07%	5.36%
Poor Health Rate	4.75%	1.55%
Accept 2014 Medicaid Expansion	31 = Yes	19 = No

The zero-order correlation between 2012 racial resentment and lesser acceptance of responsibility for marketplaces was significant (r = -.54 p < .01). In regression (Table 2) racial resentment was again significantly associated with less marketplace acceptance (b = -17.73, B = -0.43, Robust SE = 5.39, p < .01), as was not accepting Medicaid expansion (b = -0.93, B = -0.37, Robust SE = 0.28, p < .01). Overall, the model successfully predicted acceptance of responsibility for marketplace (Model R^2^ = 0.50, p < .01).

**Table 2:** States’ Marketplace Acceptance Regressed on Racial Resentment, Conservatism, Uninsurance, Health, Medicaid Expansion Acceptance

	b	Beta	Robust SE	95%CI
**Intercept**	13.58**		3.20	7.13 20.04
**Racial Resentment**	-17.73**	-0.43	5.39	-28.59 -6.87
**Conservatism**	-0.04	-0.17	0.04	-0.11 0.04
**Uninsurance**	0.01	0.02	0.03	-0.04 0.06
**Poor Health**	0.07	0.09	0.1	-0.13 0.27
**Accept Medicaid Expansion**	-0.93**	-0.37	0.28	0.38 1.48

R^2^ = 0.50**; *p < .05 **p < .01

The zero-order correlation between 2016 racial resentment and lower plan selection rates was significant (r = -.26 p < .05). In regression (Table 3), racial resentment was significantly associated with lower plan selection rates (b = -170.49, 63.74, B = -0.42, Robust SE = 63.74, p < .01). Conservatism (b = -1.11, B = -0.57, Robust SE = 0.36, p < .01) proved correlated with lower plan selection rates and not accepting Medicaid expansion (b = - 3.73, B = -0.31, Robust SE = 1.77, p < .05) and poor health (b = 2.16, B = 0.30, Robust SE = 0.98 p < .05) were associated with higher acceptance rates. The model successfully predicted higher consumer acceptance rates (Model R^2^ = 0.38, p < .01).

**Table 3:** States Plan Selection Rates Regressed on States’ Racial Resentment, Conservatism, Uninsurance, Health, Medicaid Expansion Acceptance, Marketplace Acceptance

	b	Beta	Robust SE	95% CI
**Intercept**	181.47**		40.52	99.75, 263.19
**Racial Resentment**	-170.49**	-0.42	63.74	-299.03, -41.95
**Conservatism**	-1.11**	-0.57	0.36	-1.83, − 0.39
**Uninsurance**	0.33	0.16	0.26	-0.20, 0.86
**Poor Health**	2.16*	0.30	0.98	0.19, 4.13
**Accept Medicaid Expansion**	-8.32*	-0.39	3.49	-15.36, -1.28
**Accept Marketplaces**	-3.73*	-0.31	1.77	-7.30, -0.16

R^2^ = 0.38** ; *p > .05 **p > .01

### Sensitivity analysis

Measured in 2016 instead of 2012, racial resentment once more was significantly associated with states’ lesser acceptance of responsibility for marketplaces (*b* = -18.62, B = -0.45, Robust SE = 4.17, p < .01), as was rejecting Medicaid expansion (b = -0.86, B = -0.37, Robust SE = 0.26, p < .01). Measured in 2012, three years before the second enrollment period, racial resentment again was associated with lower plan selection rates as a nearly significant trend (*b* = -144.15, B = -0.38, Robust SE = 74.82, p < .06). Not accepting Medicaid expansion (*b* = -9.20, B = -0.40, Robust SE = 3.62, p < .01), poor health (b = 2.14, B = 0.29, Robust SE = 1.03, p < .05), and Conservatism (*b* = -1.05, B = -0.57, Robust SE = 0.36 p < .01) also were associated with plan selection rates.

Estimating marketplace responsibility with GLM with a Probit link, racial resentment in 2012 was again significantly related to assuming less responsibility for marketplaces (*b* = -23.96, SE = 8.86, p < .01). Not accepting Medicaid expansion (*b* = -1.76, SE = 0.62, p < .01) also was significantly related to state responsibility for marketplaces. After logarithmic transformation of plan selection rates, racial resentment in 2016 was still significantly associated (*b* = -1.84, B = -0.45, Robust SE = 0.68, p < .01) with the outcome. Not accepting Medicaid expansion (*b* = -0.10, B = -0.39, Robust SE = 0.04 p < .01), poor health b = 0.03, B = ,35, Robust SE = 0.01,p < .05) and Conservatism (*b* = -0.01, B = -0.55, Robust SE = 0.00 p < .01) too were significantly associated with the outcome.

## Discussion

Racial resentment was greater in states minimizing responsibility for marketplaces’ administration and operation than in eagerly participating states. States higher in racial resentment also showed lower rates of plan enrollment, pointing to a racial component’s presence when states displayed less commitment to implementing marketplace provisions and fulfilling the ACA’s coverage-improvement and equity-promoting missions. Differences persisted after controlling for differences in conservatism, uninsurance, poor health, and rejection of Medicaid expansion and proved robust to alternate specification and estimation approaches.

Along with greater racial resentment, states rejecting Medicaid expansion experienced more enrollment in marketplace plans. This outcome likely occurred because expansion-rejecting states had a larger pool of persons who were eligible for subsidized purchase of private coverage from exchanges because they did not receive expanded Medicaid. Coverage was mandatory during years covered by the study and non-expansion state marketplaces were assisting people denied expanded Medicaid in meeting their ACA responsibilities.

Conservatism also proved associated with lower plan selection rates. Conservatives might have embraced marketplaces and marketplace-sold private coverage because, unlike publicly supported Medicaid, marketplaces operate in the private sector. They even enjoy a conservative pedigree: historically, for increasing health insurance coverage, marketplaces were considered a vehicle for bringing consumer choice and insurer competition to bear [1]. Furthermore, seeking to maximize state over federal power [53], conservatives might have a preferred state-operated marketplaces [7]. As events unfolded, conservative states’ ACA objections appear to have overridden their conservative philosophical commitments.

Conservatism’s and Medicaid expansion rejection’s independent influences demonstrated in this study highlight how anti-welfare stances [7] have sources beyond racism. Akin to the present state-level findings Gilens [54] demonstrated, after controlling for individuals’ explicit race-based disapproval, conservative values predicted opposition to welfare spending. Similarly, Whites oppose funding for treatment and harm reduction interventions which are perceived to enable addictive lifestyles and, undermining claims of racism, African Americans oppose them too [55]. Racial resentment continues to be very important, but its role should be further understood in a context of competing explanations.

Better understanding is needed of policies and administrative practices that promote reaching out and enrolling eligible persons and creating systems favorable to doing so, placing racial resentment’s role in contexts of sociopolitical and administrative decision-making. Implicit and explicit bias [56–59] and racial resentment can infuse public and official environments: specific pathways should be identified from ingrained cultural beliefs that African American do not deserve public support to insurance marketplace outcomes. Questioning is needed as to how racial resentment—infused throughout popular, policy makers,’ and health administrators’ culture—influences decisions to seek distance from marketplaces and implement them reluctantly. Efforts are not destined to be futile: that there can be greater acceptance is demonstrated by Medicaid expansion’s increasing acceptance from 31 to 40 states.

Marketplaces have been overlooked in many assessments of the ACA, although they brought generous coverage to more than 15 million people who otherwise might have gone without. And marketplaces were especially important for African Americans: more African Americans were eligible for subsidized coverage than for expanded Medicaid in 2014 and private coverage disparities subsequently declined [60]. Yet, research literature reflects a widely shared imbalance in attention: whereas more than 27 published studies concerned disparity reduction in Medicaid expansion [61], yet only one concerned racial disparity reduction through marketplace purchases [60].

Overcoming challenges to marketplace success should be a high priority concern. Marketplaces are difficult to implement, as bureaucratic and enrollment complexities present formidable barriers. Immediately before ACA implementation, for example, 50% of uninsured potential marketplace consumers were unfamiliar with basic insurance concepts and terms [62]. Meeting this challenge, marketplaces presented standardized, structured information, and employed navigators and other assistors to assist consumers in reviewing coverage options, completing applications, and appealing marketplace decisions (Norton et al., 2014).

That marketplaces would close disparities was hardly assured. Disparities in take-up of federal health insurance benefit programs occur often [63] due to administrative burdens discouraging African Americans and other vulnerable groups especially [64]. African Americans’ distrust of healthcare systems [65], aversive experiences with health programs [66], and mistrust of government fairness [67] foretold limited African American uptake. On the other hand, paradoxically, Whites’ ACA disapproval might have led them to miss this opportunity for coverage even more than Blacks. Ultimately, both groups gained in coverage and African Americans gained more than Whites [60].

African Americans’ disproportionate coverage gains arose partly because of greater eligibility for subsidies, but also because some states aggressively targeted African American and other minority communities to prevent expected marketplace inequalities from materializing. Beyond minimum requirements [34], officials in these states aggressively sought to raise awareness of coverage possibilities and requirements and facilitated plan selection by extensively using brokers and navigators to support consumer enrollment [68]. Drawing lessons from these experiences, federal requirements can be rewritten to encourage implementation of more African American sensitive marketplaces. Under new federal guidelines, racial resentment can be countered and the ACA’s avowed equity aims [8] can be advanced.

### Limitations

These results demonstrate empirical associations, but they cannot prove causal connections. When studying individuals, studies have shown that attitudes like racial resentment can sometimes *cause* negative ACA stances. One investigator [9] randomly assigned survey respondents to receive questions proposing government health plans framed as “President Obama’s proposal” or “President Clinton’s proposal.” More respondents showed resentment when the proposal was framed as Obama’s. Although random assignment of states is infeasible, observational approximations have improved and are ripe for application. For the present study, however, unmeasured covariates are possible, and causation remains plausible but undemonstrated.

Further, plan selection is an initial stage in a process that includes payment (“effectuated enrollment”) and discontinuation, reenrollment, or switching, assessment of which exceeds the scope of the present study. Future research studies can provide a comprehensive account of enrollment processes. Additionally, though this study rests on state estimates of racial resentment employing state-of the art methods, estimation accuracy error likely would reduce the ability to detect true findings and represents a conservative source of error. Recent proposals argue for the reformulation racial resentment with transformed measurement.

Finally, using a retrospective cross-sectional approach, this study fails to capture the evolving landscape of marketplace operations nationwide. Some states’ marketplace structural decisions changed as a few states reconsidered original decisions, and the wider policy environment shifted somewhat since early years covered in the study. A national individual mandate enforced by a penalty was in effect in 2015 and lifted in 2019, but it affected marketplace success less than feared [69]. Additional changes were brought on by the COVID-19 pandemic. ACA antipathy remained strong throughout, as was documented previously, and it even drove some of these developments. Racial resentment likely remains a continuing force in marketplace implementation decision making.

## Conclusion

Despite these limitations, findings from the present study suggest that state-level racial resentment is a correlate of marketplace decision making and marketplace success. Especially in a highly politicized environment accompanying the ACA and continuing to the present, racial resentment can infuse state political cultures and be implicated in decisions about how to organize marketplaces—with consequences indicating greater or lesser success. As individuals’ racial resentment is associated with beliefs and actions, so are state cultures’ varying levels of racial resentment influence ACA policy decisions and policy success.

## Data Availability

State marketplace variables are publicly available from the 2015 National Conference of State Legislators Report and can be found at https://nationaldisabilitynavigator.org/wp-content/uploads/news-items/THI_marketplaces_diverse_populations_july_2015.pdf. State rates of marketplace plan selection are publicly available from the Kaiser Family Foundation at https://www.kff.org/health-reform/state-indicator/marketplace-enrollment/?activeTab=graph&currentTimeframe=0&startTimeframe=9&sortModel=%7B%22colId%22:%22Location%22,%22sort%22:%22asc%22%7D. Data used to assess state racial resentment are not publicly available due to research identifiability but are available from the corresponding author on reasonable request.
